# Metabonomic Study on the Antidepressant-Like Effects of Banxia Houpu Decoction and Its Action Mechanism

**DOI:** 10.1155/2013/213739

**Published:** 2013-10-23

**Authors:** Zhanqiang Ma, Weiwei Ji, Rong Qu, Mingyan Wang, Wen Yang, Zhen Zhan, Qiang Fu, Shiping Ma

**Affiliations:** ^1^Department of Pharmacology for Chinese Materia Medica, China Pharmaceutical University, Nanjing, Jiangsu 211198, China; ^2^Discipline of Chinese and Western Integrative Medicine, Nanjing University of Chinese Medicine, Nanjing 210046, China

## Abstract

The aim of this study was to establish an experimental model for metabonomic profiles of the rat's brain and then to investigate the antidepressant effect of Banxia Houpu decoction (BHD) and its possible mechanisms. Behavioral research and metabonomics method based on UPLC-MS were used to assess the efficacy of different fractions of BHD on chronic unpredictable mild stress (CUMS) model of depression. There was a significant difference between the BHD group and the model group. Eight endogenous metabolites, which are contributing to the separation of the model group and control group, were detected, while BHD group regulated the perturbed metabolites showing that there is a tendency of recovery compared to control group. Therefore, we think that those potential metabolite biomarkers have some relationship with BHD's antidepression effect. This work appraised the antidepressant effect of Banxia Houpu decoction as well as revealing a metabonomics method, a valuable parameter in the TCM research.

## 1. Introduction

Major depressive disorder (MDD), also called major depression, a highly debilitating and widely distributed illness in the general population and an incapacitating disorder characterized by depressed mood, anhedonia, and altered cognitive function that causes a heavy burden to patients and their families, as well as to society [1]. The lifetime prevalence of MDD is approximately 17% of the population and results in tremendous secondary costs to society [2, 3]. There are typical antidepressant drugs, including selective 5-serotonin reuptake inhibitors (SSRIs), serotonin-noradrenergic reuptake inhibitors (SNRIs), tricyclic antidepressants (TCAs), and other atypical antidepressants such as monoamine oxidase inhibitors (MAOIs) in clinical treatment [4]. However, the effects of these antidepressants are often inconsistent and many of them often cause side effects, such as apathy, sedation and cognitive disorders, sleep disorders, and sexual dysfunction [5]. Therefore, there is an urgent need for new effective and better tolerable antidepressants.

In the verification of antidepressant drugs, CUMS is considered to be a well-assured animal model [6, 7]. Many behavioral and biochemical changes induced by CUMS are reversible by antidepressant treatments [8]. Therefore, the CUMS model is suitable for investigating the pathophysiology of depression and antidepressant effects of diverse drugs [9]. Metabonomics can be interpreted as a value of alterations in metabolism in biological fluids and tissues of the organism [10, 11], which is a new aspect for elucidation of metabolic reactions of biological systems of any circumstantial impulses [12]. Global metabolites profiling of biofluids has been assigned in the fields of drug discovery, therapeutic scrutinizing, and assessment of drug effectiveness and toxicity [13–16]. 

Banxia houpu decoction (BHD) is a traditional Chinese herbal prescription, which was first recorded in Jilicn Gui Yao Lue in the Han Dynasty in Chinese history (202BC-220AD). Its recipe is composed of five herbal medicines, *Pinellia ternata *Breit, *Magnolia officinalis* Rehd. et Wils, *Poria cocos* Wolf,* Perilla frutescens* Britt, and *Zingiber officinale*, and is designed for the treatment of liver qi stagnation and phlegm accumulation, in the aspect of traditional Chinese medicine (TCM) [17], and is mainly empirically advocated for the treatment of mental diseases including depression and other disorders [18].

Even though BHD has been applied frequently in Chinese hospitals for many years, the antidepressant researches of BHD were mainly concentrated on behavioral research. Therefore, its definite knowledge is still vague [19]. In addition, little is known about the changes of the whole metabolites in an organism treated with BHD. Therefore, we evaluated the antidepressant action of the decoction in rats to get a better understanding of this empirical formula. In light of this, we performed a UPLC-Q-TOFMS metabonomic approach to characterize the global metabolic profiling of rats brain homogenates in antidepressant studies of BHD to evaluate the pharmacological effect of BHD on CUMS. Meanwhile, some significantly changed metabolites were used to explain the mechanism.

## 2. Methods and Materials

### 2.1. Materials and Reagents

Banxia Houpu decoction (*Pinellia ternata *Breit, *Magnolia officinalis *Rehd. et Wils, *Poria cocos *Wolf, *Perilla frutescens* Britt, and *Zingiber officinale*) was purchased from Yifeng drug store (Nanjing, China) and was authenticated according to the standards documented in Chinese Pharmacopoeia by Minjian Qin, a pharmacognosist in our team. 

### 2.2. Preparation of Banxia Houpu Decoction

*Pinellia ternata* Breit (~12 g), *Poria cocos* Wolf (~12 g), *Magnolia officinalis* Rehd. et Wils (~9 g), *Perilla frutescens* Britt (~6 g), and *Zingiber officinale* (~15 g) were accurately weighed and mixed. These herbs were immersed in 8 times volume of water for 1 h. According to the methods of Sun et al. [20], medicinal materials were decocted twice at boiling temperature for half an hour, and then the decocted liquids were centrifuged at 3000 rpm for 5 min. The supernatant was considered as the Banxia Houpu decoction and it was concentrated and then dried in a vacuum oven at 55°C. The yield of dried powder (14.7 g) is equivalent to the 100 g of original dry materials. The final dried powder of BHD was dissolved in distilled water and finally prepared the 1.8 g/mL of BHD. The sample was stored at 4°C. The doses of distilled water extract of BHD were expressed as gram of the original dry materials per kilogram body weight, and the dose (6 g/kg) of distilled water extract of BHD for animals was converted from the human dose.

### 2.3. Animals and Drug Administration

Male Sprague Dawley rats weighing 180–220 g were purchased from the Experimental Animal Centre of China Pharmaceutical University. The animals were housed in polypropylene cages under standard experimental conditions of room temperature (20 ± 2°C), humidity (50 ± 10%) and light (12-h light/dark cycle, lights on at 7:00 a.m.). Animals were acclimatized for 7 days before any experimentation. All experiments and animal care were performed in compliance with the National Institute of Health Guide (NIH publication no. 80-23, revised 1996) and the PR China legislation for the care and use of laboratory animals. The doses of distilled water extract of BHD were expressed as gram of the original dry materials per kilogram body weight, and the dose (6 g/kg) of distilled water extract of BHD for animals was converted from the human dose.

The animals were divided into three treatment groups as follows: vehicle-control (0.9% physiological saline), vehicle-CUMS (0.9% physiological saline), and CUMS-BHD treatment (6 g/kg). All drugs were intraperitoneally (i.p.) administered once during the last three weeks of the CUMS procedure.

### 2.4. Chronic Unpredictable Mild Stress (CUMS) Procedure

**Figure 1:**
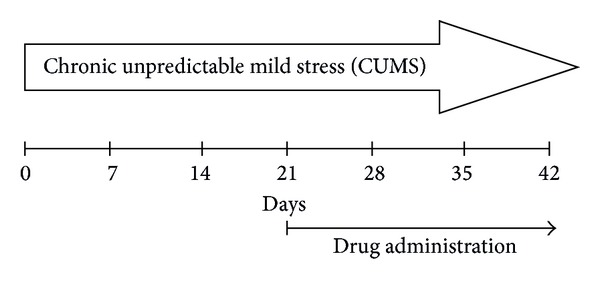
Experimental manipulations rats during the present study.

The CUMS procedure was adopted as described by Willner et al. [21] with slight modifications. The following stressors (10) were used to provoke depressive states: cage tilt (45°, 23 h); soiled cage (100 mL of water spilled onto the bedding (23 h)); withdrawal of food or water (23 h); continuous overnight brilliance; cold water swimming (4°C for 5 min); empty water bottles (23 h); swing on the rocking bed (200 Hz for 5 min; HY-4A, Shunhua Scientific instrument limited Company, China); 2 h behavioral hindrance in a tube (diameter: 8 cm, length: 20 cm); sporadic illumination (light on and off every 2 h). Each animal was exposed to one stress per day individually for 6 weeks. The whole experiment was conducted for 6 weeks, and the procedural succession was as follows: (1) stressors induction: 1–42 days; (2) drug administration: 22–42 days; (3) 1% sucrose utilization test: 40 days; (4) open-field behavior test: 41 days; (5) forced swim test: 42 days (Figure 1).

### 2.5. Behavior Test

#### 2.5.1. Forced Swim Test

The forced swim test (FST) method was similar to that described by Porsolt and colleagues with minor modifications [22]. Rats were kept in 30 cm of water contained in glass cylinders separately (20 cm in diameter, 50 cm deep), and maintained at 22 ± 1°C. Each animal was forced to swim for 6 min, and the total duration of immobility was measured during the last 4 min. The definition of immobility was the absence of all movements only with motions required to maintain the animal's head above the water. Observers were blind to the group treatment of the rats.

#### 2.5.2. Open-Field Test

The open-field test was carried out on day 41 between 8:00 am to 12:00 am in a quiet room as previously explained (≤60 dB) [23]. The open-field apparatus was a four-sided 100 cm × 100 cm × 40 cm wooden enclosure, with a white-painted floor, and separated by 25 equal squares with black lines and sidewalls painted black. Tests were performed in a darkened place lit by two 60-W light bulbs which were hanged over the center of the open field. Each rat was placed individually into the center of the arena and permitted free exploration. The numbers of squares crossed by the rats (crossings) and of standing on the hind legs (rearings) were recorded during a test of 4 min. This apparatus was cleansed with a detergent and dried after occupancy by each rat.

#### 2.5.3. Sucrose Preference Test

A sucrose preference (SP) test [21, 24] was applied to operationally define anhedonia [25]. Briefly, 72 h before the test, rats were trained to adapt to 1% sucrose solution (w/v). Two bottles of 1% sucrose solution were placed in each cage, and 24 h later 1% sucrose in one bottle was replaced with tap water for 24 h. At the end of adaptation, rats were deprived of water and food for 24 h, followed by the sucrose preference test, in which rats were housed in individual cages and had free access to two bottles with 200 mL of sucrose solution (1%, w/v) and 200 mL of tap water, respectively. All the procedures were performed in the same cage with minimum disturbances. The SP test was repeated after 40 days of CUMS. The SP was calculated according to the following equation: SP = sucrose intake/(sucrose intake + water intake) × 100.

### 2.6. Collection and Preparation of Brain Sample

Three rats from each group were decapitated 60 min after the behavioral tests. Brains were removed carefully and quickly stored in liquid nitrogen. The brain tissues were weighed accurately and homogenized in ice-cold methanol (each 1 g brain tissue was mixed with 4 mL methanol). Precisely drawn 1 mL of homogenate was centrifuged at 13,000 rpm for 10 min; the supernatant was decanted and evaporated to give the dry powder at 37°C under a gentle stream of nitrogen. The dried residue was then reconstituted in 100 *μ*L of acetonitrile-water (10: 90, v/v). After it was vortexed for 30 s, the content was transferred to 2 mL glass vials and an aliquot of 5 *μ*L was injected for UPLC-MS/MS analysis.

### 2.7. Liquid Chromatography

UPLC was performed on a Waters Acquity UPLC system (Waters, Milford, MA, USA), consisting of a binary solvent delivery system, an on-line degasser, an autosampler, and a photodiode array detector (PDA) system. An ACQUITY UPLCTM BEH C_18_ (2.1 × 100 mm I.D., 1.7 *μ*m, Waters, Milford, USA) column was used for all the analyses. The mobile phase composed of A (acetonitrile) and B (0.1% formic acid, v/v) with a gradient elution 0–11 min, 5–90% A. The flow rate of the mobile phase was 0.5 *μ*L/min, and the column autosampler temperatures were maintained at 30 and 10°C, respectively.

### 2.8. Mass Spectrometry

The MS analysis was performed on a Waters ACQUITY Synapt Q-TOF mass spectrometer connected to the Waters Acquity UPLC system via an electrospray ionization interface (ESI). High purity nitrogen was used as the nebulizer and auxiliary gas and argon was used as the collision gas. The Q-TOF mass spectrometer was operated in positive ion mode with a capillary voltage of 3 kV, a sampling cone voltage of 30 V, a cone gas flow of 50 L/h, a desolvation gas flow of 600 L/h, a desolvation temperature of 350°C, a source temperature of 120°C, a collision energy of 6 V, and the full scan spectra from 100 to 1000 Da.

### 2.9. Data Analysis

Each sample was represented by a total ion current (TIC) chromatogram. The UPLC-MS raw data were processed using the Marker Lynx Applications Manager version 4.1 (Waters Corp. Milford, USA). The raw data were transformed into a single matrix containing aligned peaks with the same *m*/*z* retention time pair along with normalized peak intensities and sample name. The three-dimensional data, peak number (RT-*m*/*z* pair), sample name, and normalized ion intensity were introduced to SIMCA-P 11.0 software package (Umetrics, Umea, Sweden) for principal component analysis (PCA) and partial least squares discriminant analysis (PLS-DA). Data in the figures or the table are expressed as mean values ± SD. The differences between groups were analyzed by one-way ANOVA or Student's *t*-tests, with *P* < 0.05 considered as significant.

## 3. Results

### 3.1. Analysis of Behavior Test

#### 3.1.1. Forced Swim Test

**Figure 2:**
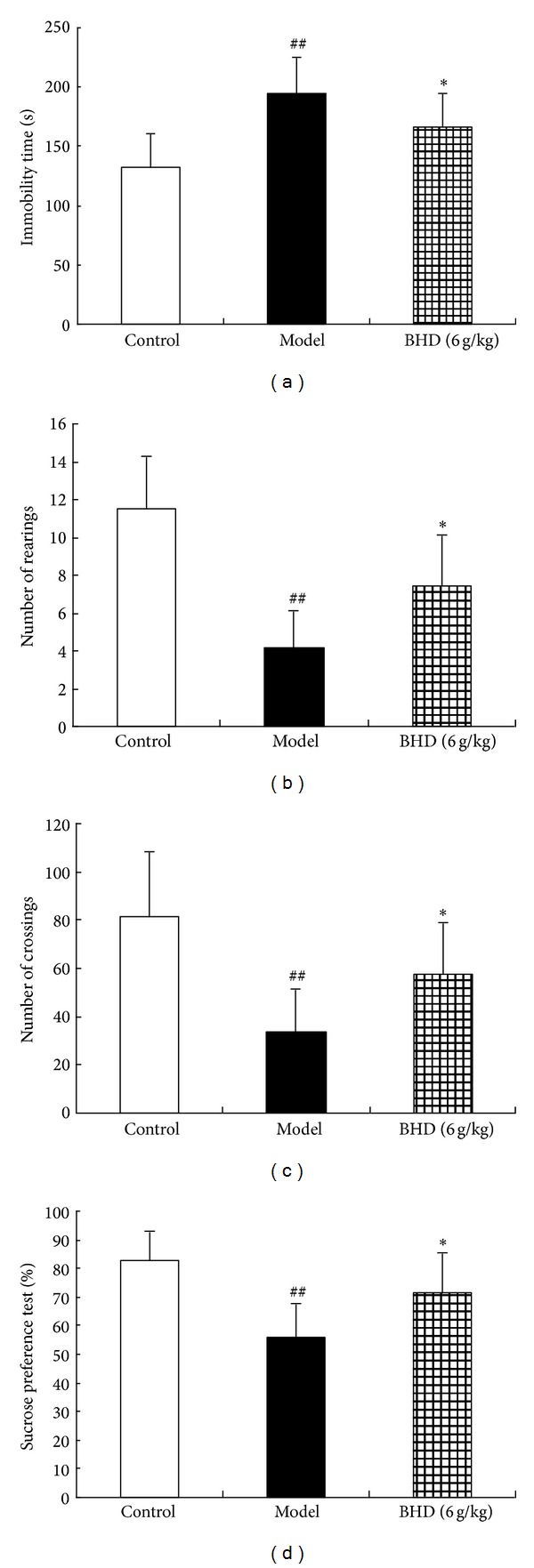
(a) Effect of BHD on forced swimming test after six weeks of CUMS. Each column represented as the mean ± SEM (*n* = 10–12). ^##^ *P* < 0.01 as compared with control group; *P < 0.05, compared with model group. (b) and (c) Effects of BHD on locomotor activity of rats after CUMS. (b) Number of rearings during the 5 min session. (c) Number of crossings during the 5 min session. Each column represented as the mean ± SEM (*n* = 10–12). ^##^ *P* < 0.01 as compared with control group; *P < 0.05, compared with model group. (d) Sucrose preference of rats after six weeks of CUMS. Percentage of sucrose preference was measured for a period of 1 h, after the 23 h food and water deprivation. Each column represented as the mean ± SEM (*n* = 10–12). ^##^ *P* < 0.01 as compared with control group; *P < 0.05, compared with model group.

After 42-day UCMS, the ANOVA test showed a significant effect on groups for forced swim test (*F* (2,27) = 11.6002, *P* < 0.01).  Figure 2(a) shows the effects of oral administration of BHD (6 g/kg) on immobility time in the FST. Chronically stressed rats exhibited a significant increase in immobility time as compared to model animals. The statistical analysis results showed that BHD at a dose of 6 g/kg markedly decreased the immobility time (*P* < 0.05 versus model group). 

#### 3.1.2. Open-Field Test

After 41-day UCMS, the ANOVA test showed a significant effect on groups for crossings (*F* (2,28) = 13.5326, *P* < 0.01) and rearings (*F* (2,28) = 9.8743, *P* < 0.01). The locomotor activity in the CUMS experiment was observed in the open-field test (Figures 2(b) and 2(c)). The statistical analysis results showed that CUMS led to a pronounced reduction in the number of crossings (*P* < 0.01) and rearings (*P* < 0.01). By the treatment of BHD (6 g/kg), the behavioural changes for the number of rearings and crossings were significantly reversed (*P* < 0.05, *P* < 0.05, resp.).

#### 3.1.3. Sucrose Preference Test

Figure 2(d) shows the effect of BHD treatment on the sucrose preference in the control and the CUMS-treated rats. After 40 days of CUMS, the ANOVA test showed a significant effect on groups for sucrose preference (*F* (2,28) = 12.3124, *P* < 0.01). The post hoc test revealed that sucrose preference in the stress group was significantly reduced compared with the control group (*P* < 0.01). Simultaneous administration with BHD (6 g/kg) while the rats were exposed to CUMS significantly increased the percentage of sucrose consumption as compared with the model group (*P* < 0.05).

### 3.2. Pattern Recognition and Identification of Potential Biomarkers

**Figure 3:**
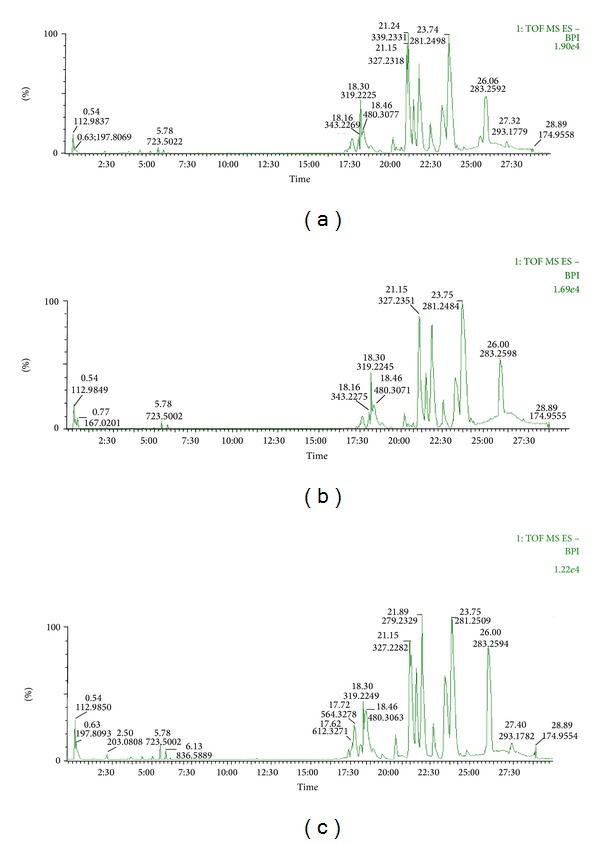
Typical base peak intensity (BPI) chromatograms obtained from rats brain (a) control group, (b) model group, and (c) treatment group in positive mode.

**Figure 4:**
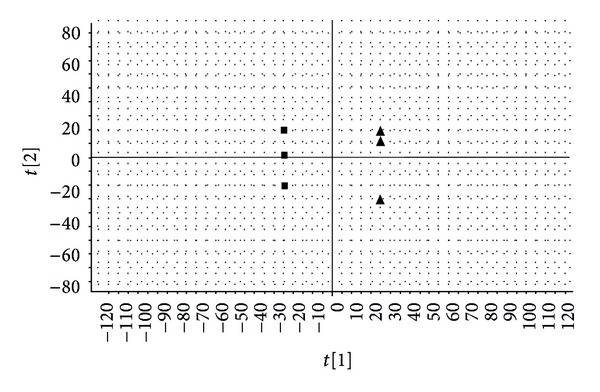
Score plot (■) control group rats, (▲) model group rats in positive ion mode from a PCA mode.

**Figure 5:**
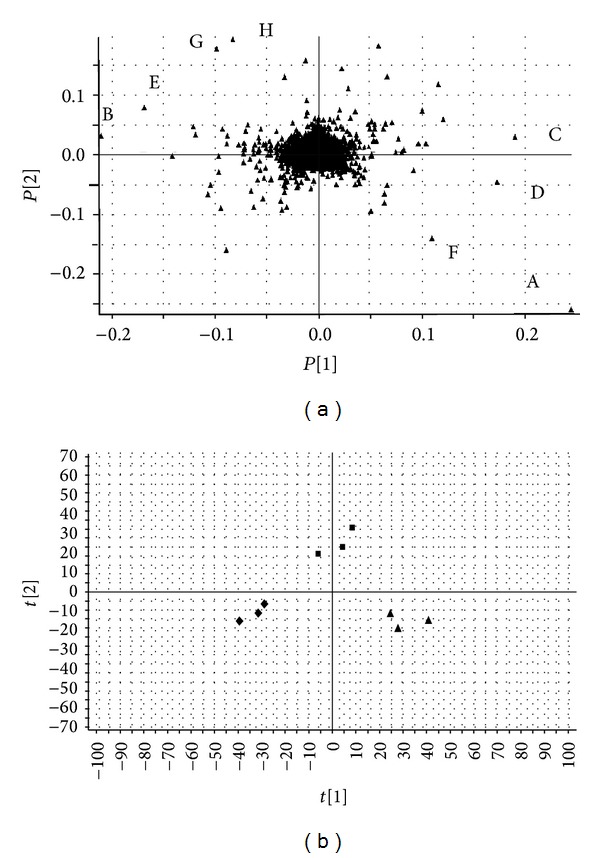
(a) Loading plot in positive ion mode from a PCA model. (A) lysoPC (18 : 0); (B) lysoPC (16 : 0); (C) PG (16 : 0/18 : 0); (D) behenic acid; (E) 8,9-epoxyeicosatrienoic acid; (F) stearic acid; (G) glycine; (H) glutamic acid. (b) Score plot (**▲**) control group rats, (**■**) model group rats, and (**◆**) treatment group rats in positive ion mode from a PLS-DA model.

**Figure 6:**
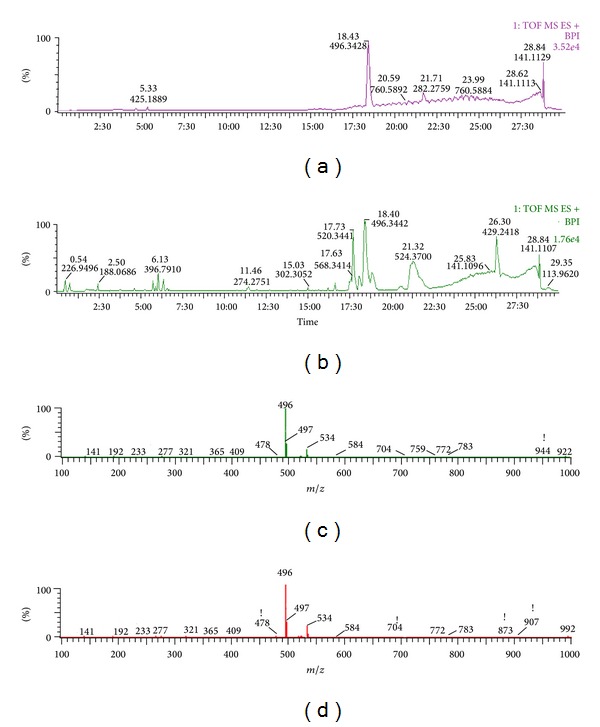
Product ion spectrum of biomarkers at *m*/*z* 520.1 in positive ion mode.

Figures 3 and 4 show the PCA score plot of characteristic UPLC/MS base peak intensity (BPI) chromatograms of rat brain tissue from all the groups demonstrating the distribution among control and model group in two aspects. Obvious separation between the control and model groups suggests that biochemical perturbation was significant in model group. The corresponding loading plot used to identify biomarkers is shown in Figure 5. The ions furthest away from the origin contribute significantly to the clustering of the two groups and may be regarded as the potential biomarkers of chronic unpredictable mild stress-induced depressant. *t*-test was used to reveal the significant differences of identified metabolites between the model and control group. The significant variables detected in the positive ion mode are summarized in Table 1. Eight endogenous metabolites (lysoPC (18 : 0); lysoPC (16 : 0); PG (16 : 0/18 : 0); behenic acid; 8,9-epoxyeicosatrienoic acid; stearic acid; glycine; glutamic acid) were tentatively identified by comparing with authentic standards or based on their molecular ion information and corresponding fragments of product ion. The biomarker with retention time and *m*/*z* pairs of 18.40–496.3391 was identified as lysoPC (16 : 0). We used it as an illustration to demonstrate the identification process. Under the same UPLC-QTOFMS conditions, we compared standard lysoPC (16 : 0) with sample and we found that peak 18.40–496.3391 and standard have the same RT (Figure 6), so the biomarker at *m*/*z* = 496.3391 was identified as lysoPC (16 : 0).

To determine whether the BHD could influence the metabolic pattern of depression in rats, a PLS-DA model was constructed. Figure 5 shows the PLS-DA score plot of three groups in positive ion mode. From the data of Figure 5, after 21 days of BHD treatment, the treatment group showed better improvement which is more similar to the control group than model group in the direction of the first principal component, which implied that BHD has intervened the metabolic process of depressed animals to some extent.

## 4. Discussion

In the present study, we investigated the metabolic pattern induced by CUMS and the influence of BHD. The CUMS model of rats successfully copied the state of depression by increasing immobility time in the FST and reduction of sucrose intake. The behavior results demonstrated the antidepressant effect of Banxia Houpu decoction.

Subtle changes could be found using a pattern recognition approach, such as PCA and PLS-DA. PCA and PLS-DA are the two most popular pattern recognition methods to gain information for classification and to identify metabolites. PCA, an unsupervised method, is applied as the first step in the separation procedure to filter out the noise, and it reduces the dimension of data to widen the observation. PLS-DA, a supervised method, which has the similar principle with PCA, is used to enhance the classification performance [26].

Eight endogenous metabolites, including amino acids, organic acids, and fatty acids, were detected, and they contributed to the separation of the model group and control group. Lysophosphatidylcholines (lysoPCs, LPC) are a class of compounds that have a constant polar head and fatty acyls of different chain lengths, position, degrees of saturation, and double bond location in plasma. LPC level can be a clinical diagnostic indicator that reveals pathophysiological changes [27]. Lysophosphatidylcholines are products or metabolites of phosphatidylcholines (PCs), which are structural components of animal cell membranes. 

Prostaglandin (PG) exists widely in many tissues. In vivo, prostaglandins (PGs) are synthesized by arachidonic acid. Prostaglandin (PG) has complex regulatory effects on the immune system as it can play a positive and positive role in immune regulation. Studies have found that PG plays an important role in neuronal oxidative damage by EP2, which promotes the inflammatory reaction around neurons [28]. More and more evidence indicates that inflammatory processes may play important roles in the pathogenesis of depression [29, 30]. Our study found that CUMS could increase prostaglandin levels in the rats. The level of prostaglandin in model rats can be turnedover by BHD. As a result of that, we inferred that BHD exerts antidepressant-like effect via regulating the PGs level.

Glutamic acid, an excitatory neurotransmitter in the mammalian nervous system [31], was significantly decreased in model group in present study, while glycine, the inhibitory neurotransmitter, was increased in model group. Studies of pathophysiology of depression and neuropharmacology found that patients with affective disorders in glutamate system abnormalities and glutamic acid conversion rate have changed in localized brain regions. The results suggested that the glutamic acid and glycine biosynthesis were affected after CUMS treatment.

Behenic acid, 8,9-epoxyeicosatrienoic acid, and stearic acid are fatty acids, in which 8,9-epoxyeicosatrienoic acid is a metabolite of arachidonic acid. Epoxyeicosatrienoic acid (EET) is epoxides of arachidonic acid biosynthesized by cytochrome P-450 (CYP450) epoxygenases. EETs function as autocrine and anti-inflammation, ion channel opening, mitogenesis and angiogenesis [32]. The change of fatty acid levels detected in this study may be caused by fatigue induced by physical stressors, which is one of the most frequently represented depressive symptoms in major depressive disorder [33]. In our study, the model group 8,9-epoxyeicosatrienoic acid was reduced, while giving BHD 8,9-epoxyeicosatrienoic acid content levels. 

Our study showed that CUMS treated BHD intervention indicated the tendency of turnover in the level of eight endogenous metabolites (lysoPC (18 : 0); lysoPC (16 : 0); PG (16 : 0/18 : 0); behenic acid; 8,9-epoxyeicosatrienoic acid; stearic acid; glycine; glutamic acid). Briefly, BHD displays a remarkable anti-depression effect by adjusting the amino acid metabolism and energy metabolism. However, further investigations are required to identify biochemical and molecular characterization to elucidate the exact antidepression mechanism of BHD.

## 5. Conclusion

A metabonomics method based on UPLC/MS was developed to establish the metabonomic profiles of rats' brain homogenate to investigate anti-depressive effect of BHD and its mechanism of action. In this work, we found eight potential metabolite biomarkers including LPCs, glycine; glutamic acid, PG; therefore, we assumed that those potential metabolite biomarkers might have some relationship with antidepressant effect of BHD. This work appraised the antidepressant effect of Banxia Houpu decoction as well as revealing a metabonomics method, a valuable parameter in the TCM research.

## Figures and Tables

**Table 1:** Identification of biomarkers on ions variations and their trend in positive mode.

RT-*m*/*z *	Biomarker identification	Metabolites	Change trend of model group versus control group	Change trend of treatment group versus model group
21.25-524.3714	A: lysoPC (18:0)	C_26_H_54_NO_7_P	↑	↓
18.10-496.3411	B: lysoPC (16:0)	C_26_H_54_NO_7_P	↑	↓
6.11-396.7923	C: PG (16:0/18:0)	C_40_H_79_O_10_P	↑	↓
21.22-340.5898	D: behenic acid	C_22_H_44_O_2_	↑	↓
18.28-319.2249	E: 8,9-epoxyeicosatrienoic acid	C_20_H_32_O_3_	↓	↑
26.01-283.2593	F: stearic acid	C_18_H_36_O_2_	↑	↓
25.77-75.0769	G: glycine	C_2_H_5_NO_2_	↑	↓
13.26-147.1317	H: glutamic acid	C_5_H_9_NO_4_	↓	↑
